# Effect of Protein Genotypes on Physicochemical Properties and Protein Functionality of Bovine Milk: A Review

**DOI:** 10.3390/foods10102409

**Published:** 2021-10-11

**Authors:** Nan Gai, Therese Uniacke-Lowe, Jonathan O’Regan, Hope Faulkner, Alan L. Kelly

**Affiliations:** 1 School of Food and Nutritional Sciences, University College Cork, T12 YN60 Cork, Ireland; 116108127@umail.ucc.ie (N.G.); t.uniacke@ucc.ie (T.U.-L.); 2 Nestlé Development Centre Nutrition, Wyeth Nutritionals Ireland, Askeaton, Co., V94 E7P9 Limerick, Ireland; Jonathan.ORegan@rd.nestle.com (J.O.); Hope.Faulkner@rd.nestle.com (H.F.)

**Keywords:** protein genetic variants, genotype frequency, milk physiochemical properties, milk functionality

## Abstract

Milk protein comprises caseins (CNs) and whey proteins, each of which has different genetic variants. Several studies have reported the frequencies of these genetic variants and the effects of variants on milk physicochemical properties and functionality. For example, the C variant and the BC haplotype of α_S1_-casein (α_S1_-CN), β-casein (β-CN) B and A_1_ variants, and κ-casein (κ-CN) B variant, are favourable for rennet coagulation, as well as the B variant of β-lactoglobulin (β-lg). κ-CN is reported to be the only protein influencing acid gel formation, with the AA variant contributing to a firmer acid curd. For heat stability, κ-CN B variant improves the heat resistance of milk at natural pH, and the order of heat stability between phenotypes is BB > AB > AA. The A_2_ variant of β-CN is more efficient in emulsion formation, but the emulsion stability is lower than the A_1_ and B variants. Foaming properties of milk with β-lg variant B are better than A, but the differences between β-CN A_1_ and A_2_ variants are controversial. Genetic variants of milk proteins also influence milk yield, composition, quality and processability; thus, study of such relationships offers guidance for the selection of targeted genetic variants.

## 1. Introduction

As the demand for milk and milk products increases continuously, and since milk provides essential nutrients in the human diet [1,2], studies on milk and dairy products have generated a lot of attention in dairy related research.

Protein is a macronutrient for the human body [1], and accounts for about 3.5% of milk mass, typically comprising approximately 80% casein and 20% whey protein [2]. Four forms of casein are found in milk protein, including α_S1_-CN, α_S2_-CN, β-CN, and κ-CN, and their genes are found at bovine chromosome 6 [3,4], coded as CSN1S1, CSN1S2, CSN2 and CSN3, respectively [1,5]. These proteins have several genetic variants, as described by Caroli et al. [6] and Farrell et al. [7]. The gene of α-lactalbumin (α-lac) in the whey protein fraction is located on bovine chromosome 5, coded as LAA [3], and that of β-lactoglobulin (β-lg) is coded by the PAEP gene (or LBG gene) [1], which is situated on bovine chromosome 11 [8]. Polymorphisms of CSN1S1, CSN2, CSN3 and PAEP have widely been studied [6,9], but only a few polymorphs of LAA and CSN1S2 have been identified, mainly in French breeds [10]. The selection of milk protein phenotypes is regarded as a practical way for altering the composition of milk protein, and traditional methods for improving milk quality included estimating the bull breeding values by the phenotypes of their numerous female offspring [10].

In this article, the effects of milk protein genetic variants on milk protein structure, milk composition, processing properties, and functionality, e.g., coagulation, foaming and emulsifying properties, are discussed.

## 2. Milk Protein Genetic Variants and Genotyping Frequency

The genetic variants of β-lg were the earliest to be identified [11], followed by the caseins [12]. Farrell et al. [7] reported that eight variants are associated with CSN1S1, from A to H, four are associated with CSN1S2 (A, B, C, D), and twelve variants are found in CSN2 (A_1_, A_2_, A_3_, B, C, D, E, F, G, H_1_, H_2_, I,) [5]. In Korean native cattle, A_4_ is found in CSN2 [13], and the I variant was characterized by Lühken et al. [14]. Twelve variants are detected in CSN3 (A, B, B_2_, C, E, F_1_, F_2_, G_1_, G_2_, H, I, J) [6,7], while, in some studies, F_1_ is regarded as F [15]; F_2_ is regarded as F by Prinzenberg et al. [16] and in GenBank no. AF123250 [6]; G_1_ is the same as G [16,17]. Eleven variants are associated with PAEP (LBG), which are A, B, C, D, E, F, G, H, I, J, W [7]. Only three variants are reported in LAA (A, B, C) [7]. Bovine milk can be homozygous when cows contain the same type of variant, or heterozygous when two different variants with allelic co-dominance are present [18].

### 2.1. Genotype Establishment and Protein Nomenclature

Reports of protein nomenclature in cows’ milk announced by the Milk Protein Nomenclature Committee have been updated in six revisions between 1960–2004, introducing the findings of protein genetic variants [7,19,20,21,22,23]. The nomenclature of the proteins is supervised by the Committee and investigators have to show conclusive evidence to prove the newly named protein is different to any previously isolated or characterized proteins [19]. To establish protein nomenclature, various techniques have been applied in recent decades for protein genetic profiling (Table 1).

Genotypes of β-lg were the first to be isolated and named among milk proteins, and it was found by Aschaffenburg and Drewry [11] that the secretion of β-lg types is genetically controlled, and they proposed that variants should be named using letters. The nomenclature of β-lg types was based on β-lg existing as two forms, which can be discerned by electrophoresis at pH 8.6 that are defined genetically [19]. β-lg variants A and B were also distinguished through their different electrophoretic mobilities at pH 4.65 by Timasheff et al. [26,27,28], where B was slower than A. β-lg-C was identified by zonal electrophoresis at alkaline pH, where it moved more slowly than β-lg-B [44]. β-lg D variant was identified later by Grosclanels et al. [45], and confirmed by Larsen and Thymann [46], Meyer [47], Michalak [48]. Later, three other variants E, F, and G, were separated from previously identified variants using starch-gel electrophoresis [11,49,50,51,52,53], and their primary structures were established by Bell et al. [50]. The H variant was separated from the B variant using isoelectric focusing-immobilized pH gradient (IEF-IPG) gel [29,30]. The W variant was separated from the A variant using chromatofocusing [54], and the I variant and J variant were identified using ion-exchange chromatography [55].

α-lac classification was firstly studied according to its biological role in the enzymatic synthesis of lactose; two forms, A and B were distinguished [56,57]. Later, the A and B variants were separated using alkaline gel electrophoresis, where B moved more slowly than A [58,59]. The C variant was found using filter-paper electrophoresis in alkaline condition, under which conditions it moved more slowly than the B variant [60].

Thompson et al. [24,25] identified three genetic variants (A, B, C) of α_S1_-CN based on their different mobilities on starch-gel electrophoresis, with mobilities of 1.18, 1.10 and 1.07, respectively, and the D variant was found to have a relative mobility of 1.14 [20]. From 1970 to 1972, some studies confirmed the primary structures of known α_S1_-CN variants A, B, C, D, which made the definition of these variants clearer [61,62,63]. The E variant was characterized using electrophoresis, where it had slower mobility than the C variant in urea alkaline gels [52,53,64]. The F variant was found by Erhardt [65] by comparing the isoelectric focusing patterns with the E variant, where the E had a more acidic isoelectric point (pI) than F. The G variant was found by Rando et al. [66,67,68], and Mahé [69] reported that the H variant showed different band on isoelectric focusing to previously identified variants. The I variant was characterized using IEF analysis and confirmed using PCR-restriction fragment length polymorphism (PCR-PFLP) [14].

Four variants of α_S2_-CN, i.e., A, B, C and D, have been identified using gel electrophoresis [23]. 

Aschaffenburg [70,71] first proposed a nomenclature for β-CN and its variants; three forms, A, B and C in samples from individual cows were separated by paper electrophoresis using 6.0M Urea at pH 7.15, which was also confirmed by Thompson et al. [72]. Knowledge of β-CN broadened in 1965–1970, the A variant was separated into A_1_, A_2_, A_3_ using gel electrophoresis in acidic conditions [73,74], and variant D was found, as its amino acid composition differed compared to previously identified variants [75]. The E variant was found in Italian Piedmont cattle in 1972 [76] and 1974 [77]. Mobilities of different β-CN variants in gel electrophoresis in alkaline or acid gels are different [78], where the mobility is A_1_ = A_2_ = A_3_ > B > D, E > C in alkaline gel with 9% cyanogum and 3.5 M urea; and C > B = D > A_1_ = E > A_2_ > A_3_ in acid gel with 10% cyanogum and 4.5 M urea. Thus, the A variants can be isolated from other variants under alkaline conditions [22]. Primary structures of these variants were established in 1972 [77,79], offering a clearer definition for them. In addition, variant A_4_ was proposed as it had lower mobility than the A_3_ variant in acid gel [60], and another variant with the same gel electrophoresis mobility as the B variant, but different peptide profiling, was named B_Z_ in 1970 [21]. The F and G variants were identified using reverse-phase high performance liquid chromatography (RP-HPLC) and the isolated components analyzed by mass spectrometry (MS), which made it easier to detect peptide differences due to mutations that were not evident using electrophoresis [39,40]. The H_1_ variant was found by its slowest mobility in acidic starch gel electrophoresis and identified using PCR [80], while H_2_ was determined by Senocq et al. [81] using LC-MS (liquid chromatography with mass spectrometry). The A_4_ variant was identified in Korean cattle breed using electrophoresis [82], and the I variant was identified by Jann et al. [83] using PCR. In addition, the I variant in β-CN was discriminated by MS analysis from A_2_ variant, which had not been noted due to unsuitable analytical methods in the past, as both I and A_2_ have the same pI (isoelectric point) [84]. 

κ-CN was found to be genetically variable using polyacrylamide-gel electrophoresis [85], and the Committee recommended naming κ-CN forms as A, B, C, etc. according to their mobilities, to be consistent with β-CN and α_S1_-CN [20]. Two κ-CN variants, A and B were confirmed using alkaline gel electrophoresis [86,87], the A variant had a greater mobility to the B variant as zero carbohydrate chain was associated to A [22], and their primary structures were established by Jollès et al. [88] and Mercier et al. [89]. Both A and B variants had multiple bands on alkaline gels including urea and mercaptoethanol [86,87]. The J variant was found to have one more positive charge or one less negative charge than the B variant, and its chromatograph on RP-HPLC showed a different pattern to the B variant [69]. The B_2_ variant was found by Gorodetskiĭ and Kaledin [90]. The C and E variants were identified by digestion with cyanogen bromide and analyzed using RP-HPLC [91], the F_1_ variant was characterized using PCR analysis [15], and the F_2_ variant was characterized by Prinzenberg et al. [16] using the same method. The G_1_ variant was found by IEF [17], and confirmed using PCR [7], while G_2_ was identified by Sulimova et al. [92]; these two G variants were both found by confirming their mutation points, as for the H and I variants [93].

Establishment of protein genetic variants discussed above is shown in Table 2; methods used to determine genotypes are listed, except where these were not clearly stated in the paper.

In several studies, frequencies of these protein genetic variants have been reported, as discussed below.

### 2.2. Genotype Frequency of β-CN

The main variants of β-CN are A_1_, A_2_, A_3_, B and C [83,94]. The A_2_ variant is regarded as the ancient original variant, while A_1_ is the product of mutation through natural selection [95,96]. It is important to note that the A_1_ variant is only found in bovine milk [95,97] and commercial bovine milk often contains both variants [98].

Genetic variant frequencies in Danish Holstein-Friesian and Jerseys cows were studied by Lien et al. [99]; A_2_ is the most common, followed by A_1_, then B, while A_3_ is the rarest. A similar prevalence was found in Norwegian Red cows, where A_2_ is the most frequent variant [100]. The prevalence of the β-CN A_2_ variant is probably due to its contribution to higher protein yield [100,101]. 

β-CN phenotype frequencies have also been reported, where its homozygous genotype A_2_A_2_ is the most frequent genotype in Estonian Cattle [102], Danish Jersey cows [103,104], and Norwegian Red cows [100], followed by its heterozygous genotype A_1_A_2_, while the A_1_A_1_, A_1_B, A_2_B, A_2_A_3_ and BB genotypes are rare [100,105]. Bobe et al. [106] reported that A_1_A_2_ is the most frequent genotype of β-CN in Finnish Ayrshire cows. 

### 2.3. Genotype Frequency of α_S1_-CN

For α_S1_-CN, the B variant is predominant in most European cows [99], and is more frequent than C, while they are both more frequent than the A variant [107]. The rare A variant is found in both American Holstein and Red Danish cows’ milk, while no genetic relationship is found between these two breeds [108], it has then speculated that A is a more ancient variant, as it arose independently [108]. The BB variant is the most frequent in α_S1_-CN, followed by BC and CC [100,109]. These results are also found in Danish Holstein and Estonian cattle, but not in Swedish Red or Danish Jersey cows [102,103,104]. In Czech cows, α_S1_-CN is found to contain only BB and BC variants, and BC is linked to higher milk, protein, and fat yields than BB [110]. 

### 2.4. Genotype Frequency of κ-CN

In most European breeds, the A variant of κ-CN is more frequent than the B variant [99,111,112], while E is the least frequent [99], and is only reported to exist at high frequency in Finnish Ayrshire cows [113]. Danish Holstein-Friesian and Jerseys cows genotyped AA and AB of κ-CN are the most common [99], while AA and BB genotypes are the most common in Norwegian Red cows [100], and AA and AE are the most frequent in Finnish Ayrshire cows [114]. BE and EE variants are rare in κ-CN, and never combine as composites with the rarest β-CN genotypes, A_2_A_3_ and BB [105]. Only the AA and BB variants are found in Czech cows, while the E variant is detected and haplotype EE is not detected [109]. 

### 2.5. Genotype Frequency of β-lg

Genotype frequencies of β-lg among breeds vary, where the A variant is more frequent than B in Holstein-Friesian cows, while B is more frequent than A in Jerseys cows [10,101,115] and Norwegian Red cows [100]. BB is more common than AB or AA in Norwegian Red cows [100], while AB is more common than AA and BB in Czech cows [109]. In Finnish Ayrshire cows, the AA variant is the rarest [114].

### 2.6. Composite Genotype Frequencies

A linkage disequilibrium between β-CN and κ-CN has been reported by Visker et al. [116], where the B and I alleles of β-CN only appear with the B allele of κ-CN, while the E allele of κ-CN only occur with the A_1_ allele of β-CN. Only seven haplotypes of β-κ-CN have been found, including A_1_A, A_1_B, A_1_E, A_2_A, A_2_B, BB, IB [116]. For the composite genotypes of β-κ-CN, A_2_A_2_-AA is more common than A_1_A_2_-AA, and these two composites are both frequent in Italian Holstein cows [105] while, in Finnish Ayrshire cows, A_1_A_2_-AE and A_2_A_2_-AA have been reported to be the most common composites [114].

For the composite genotypes of α_S1_-β-κ-CN, BB-A_2_A_2_-BB and BB-A_2_A_2_-AA are found to be highly frequent (around 23% of all the composite genotypes) compared with BB-A_1_A_2_-BE, BC-A_2_A_2_-BB and BB-A_1_A_2_-AA, the frequencies of which are only around 10% [100]. This is also found in Danish Holstein (DH) and Estonian cattle, but not in Swedish Red (SR) and Danish Jersey (DJ) cows [102,103,104,107]. The frequencies of some composite genotypes of α_S1_-β-κ-CN have been reported to have decreased over 10 to 20 years (from 1990s to 2000s) in DH cows [104,117] and in SR cows [118], where the frequency of BB-A_1_A_1_-AA has decreased from ~20% of all the composite genotypes to ~2%, and of BB-A_1_A_2_-AA dropped from ~40% to 15%. However, the frequency of BB-A_2_A_2_-AA has dramatically increased from ~9% to ~30% in DH cows [104,117], and of BB-A_1_A_2_-AE in SR cows has increased from 0% to 18% [104,118]. In DJ cows, the frequency of BB-BA_2_-AB has dropped from 20% to 6%, while that of CC-A_2_A_2-_BB has increased from less than 7% to 16% [104,117].

In ancient Nordic cows, found in the northern part of Europe, including Northern Finncattle, Swedish Mountain cows, Icelandic cows and Western Fjord cows, the C allele in α_S1_-CN, B allele in κ-CN and A_2_ allele in β-CN are the most prevalent, and the composite C-A_2_-B of α_S1_-β-κ-CN is reported to be the predominant haplotype in these cows [99]. These changes may be due to breeding goals, and they will have impacts on milk composition and technological properties of dairy products [104].

## 3. Impact of Protein Genotype on Milk Protein Structure

Protein structure and functionality are closely linked [119] and are the basis of its interaction with other milk components [120]. In product processing, some undesirable behaviours are associated with protein structures, or changes in structure during processing, such as gelling in processing equipment, or non-coagulation in milk curd processing, i.e., cheese-making [121]. 

The structures of the main proteins in bovine milk, including β-CN, α_S1_-CN, α_S2_-CN, κ-CN, α-lac and β-lg are influenced by genetic variants, as these lead to modifications of amino acid sequences [122]. These structural differences affect milk composition and quality, as well as the isoelectric points and electric charges of the proteins [7,9], and ultimately influence the physicochemical properties of milk [101]. 

For instance, variant C of α_S1_-CN is associated with smaller net charge compared to the B variant, which gives the C variant larger association constants and ultimately stronger self-association [123,124], and contributes to firmer curd in cheesemaking [125]. Variant A has most differences compared to other variants, as its residues 14–26 are deleted [125,126], it is less hydrophobic, and curd formed during cheese making with the A variant is softer [125]. The D variant of α_S2_-CN, which residues 51–59 are deleted [127], is less hydrophilic and less sensitive to Ca^2+^ than the other α_S2_-CN variants, due to the absence of one of the anionic phosphoseryl clusters [12]. β-lg, the main whey protein in bovine milk, is small, dimeric and soluble in dilute salt solutions [128]. One of the differences between the A and B variants of β-lg is a mutation site, D64G, on residues 61–67, which determines their conformations and ultimately makes the β-lg A variant less soluble, and gives better oligomerization and gelation properties [129]. The stability of its structure is influenced by pH [121], where significant changes of β-lg occur when the pH is between 6 and 8, i.e., the reactivity of the free thiol, the exposure of Glu_89_, and the opening-up of its central and ligand-binding sites [121,130,131]. 

It has been reported by Zhang et al. [132] that β-CN could hinder the chemical- or thermal- induced aggregation of proteins through association with denatured substrate proteins, by which β-CN is proven to have chaperone activity. The chaperone activity of β-CN is associated with its amphiphilic structure, as it forms oligometric micelles to prevent the aggregation of partially unfolded proteins [132,133,134]. This activity depends on protein secondary structure; proline is the basic element for the formation of polyproline-II structure [135], and thus β-CN A_2_, which contains additional prolines, has more polyproline-II helix formation and ultimately has a greater chaperone activity compared to A_1_ [136].

Proteolysis of β-CN by plasmin produces three fragments [137,138], consisting of residues 29–209, 106–209, and 108–209, named as γ_1_-CN, γ_2_-CN and γ_3_-CN, respectively [139]. β-casmorphin-7 (BCM-7) is released through the digestion of the A_1_ and B variants, by cleavage driven by elastase of the bond between peptides 66 (isoleucine) and 67 (histidine), it contains residues 60–66 of β-CN A_1_ [140,141], as a part of γ_1_-CN, whereas no hydrolysis by elastase happens for the A_2_ variant, which has a proline at position 67 [142]. However, in more recent studies, BCM-7 has been found to be released in A_2_ milk as well, but at a lower level [143,144]. This peptide has been controversially reported to be associated with milk intolerance symptom [145], cardiovascular disease [146], type I diabetes [146], autism [147], the aggravation of schizophrenia [13] and sudden infant death syndrome (SIDS) [148]. In addition, A_2_ milk has been reported to be more beneficial to human health compared to milk containing both A_1_ and A_2_ variants [149], as it improves the production of glutathione (GSH) [149], and is more digestible [5]. 

However, it has been concluded in an European Food Safety Authority (EFSA) science report in 2009 that no relationship exists between the consumption of A_1_ milk and reported illness [150], while Küllenberg de Gaudry et al. [151] reported that the correlation between the consumption of A_1_ or A_2_ milk and negative effects on human health are not significantly or clinically different, and that results of relevant studies are inconclusive due to the insufficient evidence or uncomprehensive study design. 

In addition, the substitutions at position 67 and 122 of the A_1_ and B variants exist in the hydrophobic part of β-CN, which could affect milk functionality, i.e., emulsifying properties [152]. The B variant has one or two more positive charges compared to the A_1_ and A_2_, respectively, which allows it to more easily bind with other functional proteins [152]. 

## 4. Milk Production and Milk Composition

In the dairy industry, milk yield and protein yield are two important parameters for profitability. High casein yield is positively associated with cheese yield, and a high content of κ-CN is favourable for its positive effect on milk coagulation [153]. Milk yield and protein yield are significantly affected by β-CN genotype [101], as well as fat percentage and fat yield [154], while protein content (in percentage) and casein content are affected by α_S1_-CN [154] and κ-CN genotypes [101,114,155]. 

### 4.1. The Effect of α_S1_-CN Variants on Milk Production and Composition

The effect of α_S1_-CN genotype on milk yield was reported by Van Eenennaam and Medrano [112] where the CC variant was related to high protein yield and milk yield. In Czech cows, the BC variant is associated with higher milk, protein and fat yields than the BB variant [110].

The effects of α_S1_-CN genotype on protein content, casein content and whey protein content are conflicting. It has been reported by Jakob [156] that the C variant contributes to higher casein content, and that the BC variant is associated with higher contents of protein, casein and whey protein compared to BB [157,158]. Devold et al. [159] reported the opposite result, where the BB variant is associated with higher protein, casein and whey protein contents compared to the BC variant. No effects are of α_S1_-CN genotype on the protein content, casein content and whey protein content of bovine milk have been reported [160,161,162]. 

Only a few studies have reported significant effects of α_S1_-CN genotype on fat content [154,163]. A slightly lower fat content is observed in milk with the C variant in Holstein Friesian cows [112,164], and the BC variant is associated with lower fat content compared to the BB variant in Angler cows [165].

### 4.2. The Effect of β-CN Variants on Milk Production and Composition 

The β-CN A_2_ variant is associated with higher protein yield compared to A_1_ [101,107]; the A_1_ variant is associated with higher fat content [164]. The I variant is reported to enhance protein percentage, protein yield, casein index and casein yield, as well as the contents of α_S2_-CN and κ-CN [116]. It has also been observed that the I variant is negatively correlated with α_S1_-CN, α-lac and β-lg contents [116]. Higher milk production levels are found to be associated with the heterozygotic genotype A_2_A_2_ variant, and higher fat content is found to be related to the A_1_A_1_ variant [114]. Lodes et al. [157] reported that the A_1_A_1_ variant is associated with higher protein and casein content, followed by A_1_A_2_ and A_2_A_2_ variants, and this trend is consistent with the study of Puhan [158]. While the A_1_A_1_ variant was found to be correlated with lowest whey protein content by Devold et al. [159], the lowest casein number was found to be linked to the A_1_A_2_ variant. However, no effects of β-CN genotypes on percentage protein or percentage fat were found by Famula and Medrano [166].

### 4.3. The Effect of κ-CN Variants on Milk Production and Composition

The B variant of κ-CN is associated with higher protein percentage compared to the C variant [101,114], and the E variant is correlated with a lower protein content compared to A and B variants [114]. Milk production is correlated with κ-CN genotypes, in the order AB > AA > BB [167]. The order of κ-CN genotypes as they relate to protein content is BB > AB > AA [155,156], or AB > AE > AA [159]. However, the order found by Lodes et al. [157] is opposite, i.e., as AA > AE > AB. In addition, Ikonen et al. [114] reported that the EE, AE and BE variants contributed to high milk yield but low protein percentage. The BB variant was found to be positively correlated with milk and protein production during the first lactation by Mao et al. [168].

### 4.4. The Effect of β-lg Variants on Milk Production and Composition

The AA variant is reported to be associated with favorable milk and protein production, while the BB variant is associated with high fat content [114]. The AB variant is reported to be associated with slightly higher protein and casein contents, followed by the AA and BB variants [159]. Higher casein number (percentage of nitrate in casein by total nitrogen in milk) is observed in the order BB > AB > AA and for whey protein content was AA, AB > BB [156,158,159]. 

The B variant was reported to be associated with high fat content in several studies [155,164,169], while the C variant was reported to be positively correlated with fat content in Jerseys cows [160] and Angler cows [165], and the D variant is associated with lower fat content in Brown cows [163].

### 4.5. The Effect of Composite Genotypes on Milk Production and Composition

β-CN genotypes are found to influence milk and protein yield and fat percentage more significantly than κ-CN genotypes, while κ-CN genotypes have a greater contribution to the percentage of protein [114]. The B allele of κ-CN in the haplotype of β-κ-CN thus contributes to protein percentage [107,170]. Combined with the positive effect of β-CN allele I on protein level [116], and the higher protein yield associated with allele A_2_ [101,107], haplotype I-B is a favorable variant for protein percentage [116], and A_2_-B is positively associated with milk and protein production [114,171]. Casein index is calculated as the proportion of milk protein present as casein, which is an indicator of cheese yield [172]. The haplotype I-B is also associated with higher α_S2_-CN and κ-CN contents, and casein index, while a negative association was found with α_S1_-CN, α-lac and β-lg contents [116]. 

The composites A_2_A_2_-AB, A_2_A_2_-AA and A_1_A_2_-AE of β-κ-CN are reported to be positively correlated with milk and protein production, while variants A_1_A_1_-BB, A_1_A_1_-AB and A_1_A_1_-BE are found in milk with high fat percentage [114]. High protein content was reported by Ikonen et al. [114] in milk genotyped A_1_A_1_-BB, A_1_A_2_-AB and A_1_A_1_-AB, while a low protein content was related to the A_1_A_1_-EE genotype.

For the composite genotype of α_S1_-β-κ-CN, B-A_1_-B was reported to be positively correlated with percentages of fat and protein in Holstein cows, Brown Swiss cows [107] and Finnish Ayshire cows [170], as well as in a local Italian Reggiana cows [173], but negatively correlated with milk yield [107]. Haplotype C-A_2_-B has similar effects to B-A_1_-B, and also leads to low milk yield and high protein concentration [107]. Although the B-B-A variant is rare in Holstein cows, its positive effect on fat percentage and negative effect on protein percentage were reported by Boettcher et al. [107], while another rare haplotype, C-A_3_-A, is reported to have the opposite effect [107]. 

## 5. Milk Coagulation

Milk coagulation properties, including rennet coagulation and acid coagulation properties, are the basis of cheese-making, and cheese yield and quality depend on rennet and acid coagulation properties of milk [115,153]. These properties are influenced by milk composition [100], casein micelle size [174,175], milk protein genotypes [115], milk protein content and composition [115,174], proportion of caseins and whey proteins [176], mineral and total salts contents and their distributions [115,175], as well as cow’s health status [177,178], lactation stage [179], breed [153,180], season [181] and feeding [182]. 

Rennet coagulation consists of two phases; the first phase is enzymatic hydrolysis of κ-CN, where negatively charged caseinomacropeptide (CMP, κ-CN peptide 106–169) is released into the serum phase, leading to destabilization of casein micelles [183,184]; the second phase is calcium-dependent casein aggregation and gel formation [185]. 

To define milk rennet coagulation properties, some key parameters may be measured using a Formagraph, including rennet coagulation time (RCT), curd firming time (k_20_, in min) and curd firmness (a_30,_ in mm) [186]. Gel formation can also be determined using rheology, through measurement of G’, the storage modulus, with RCT being determined from the time when G’ begins to increase [187].

Acid coagulation is achieved by decreasing milk pH to the pI of casein (~4.6), and its properties are normally defined by acid gelation time (GT), gel firmness at 30 and 60 min (G_30_ and G_60_), and acid gel firming rate in mm/min (GFR) [100].

Milk composition is an important parameter which affects milk coagulation properties [100]. Higher protein content improves a_30_, GFR and G_30_, and impairs k_20_; higher casein content has a positive effect on a_30_, GFR and G_30_, and a negative effect on k_20_ and GT; higher fat content leads to shorter RCT but produces weak acid gels, and higher lactose content is associated with better rennet and acid coagulation properties [84,100,188]. An optimal fat-to-casein ratio is also important for good milk coagulation properties [189].

Casein micelle size and fat globule size could affect milk rennet and acid coagulation properties; larger fat globule size leads to poorer acid coagulation properties, and larger casein micelles are associated with weak acid and rennet gels [100,174,190]. The beneficial effect of small micelle size on coagulation might be due to the large surface area for gel network formation [100], which leads to faster aggregation and stronger gel formation [174].

Milk coagulation properties can also be influenced by genotypes of α_S1_-CN, β-CN, κ-CN, β-lg and their composites [100,153,191,192]. 

### 5.1. Effect of α_S1_-CN Variants on Coagulation Properties 

It has been reported that the C variant of α_S1_-CN is responsible for good rennet coagulation characteristics, as it is related to high casein concentration [102,193]. The heterozygous genotype BC is more favourable for rennet coagulation, which leads to shorter k_20_ and higher a_30_ values [84,100,103], compared to homozygous genotype BB. Such different effects may be associated with casein micelle size, where the BC variant was linked to smaller micelles [84,100,159].

### 5.2. Effect of β-CN Genetic Variant on Coagulation Properties 

β-CN genotype has been reported to alter milk rennet coagulation properties [103], and is proposed to be associated with curd firmness [194]. The B variant of β-CN has been shown to be the most advantageous variant for milk rennet coagulation and cheese-making [115,191], and the A_1_ variant of β-CN is also favorable, while A_2_ variant leads to poor rennet coagulation [84,105]. The F variant, which is rare in modern cows, is associated with poor or non-coagulating properties [195].

The reason for poor coagulation associated with the A_2_ allele was proposed by Darewicz and Dziuba [152] who suggested that β-CN with A_2_A_2_ variant was more soluble and less hydrophobic at pH 6.5–6.7. Another possible reason, proposed by Day et al. [196], is that milk with β-CN A_2_A_2_ variant is associated with large casein micelles. The effect of casein micelle size on rennet coagulation properties has been found in several studies, where small casein micelle size is associated with a compact and firm gel network [197,198]. In addition, better rennet coagulation properties are found with the A_1_A_2_ variant of β-CN than the A_2_A_2_ variant [100]. 

Nguyen et al. [98] studied the effects of β-CN A_1_A_1_ and A_2_A_2_ on yogurt making; A_2_A_2_ milk had a longer gelation time and lower storage modulus compared to A_1_A_1_, and the microstructure of yogurt made of A_2_A_2_ milk is more porous, with thinner protein strands. These differences may be due to the different primary structures of β-CN, which determines its assembly and structural properties, and ultimately influences milk technical and functional properties [98]. Although the poor rennet coagulation properties of milk with β-CN A_2_A_2_ is a disadvantage in cheese-making, the weak gel could enhance digestion of yogurt, as the weaker and more porous gel can be broken down more easily by digestive enzymes under acidic conditions in the human stomach [98]. 

### 5.3. Effect of κ-CN Genetic Variant on Coagulation Properties 

Comin et al. [105] reported that κ-CN is the most important milk protein in rennet coagulation, as it is key to casein micelle stability, providing steric and electrostatic repulsion between micelles to prevent aggregation through the surface ‘hairy’ layer of micelles [115]. 

Poor coagulating and non-coagulating milk are found to be associated with low relative κ-CN content [199], which is probably due to the negative correlation between κ-CN content and casein micelle size [200]. The B variant is found to be associated with high milk quality in European cattle breeds [201] and, in comparison to the A variant, B is found to be associated with shorter rennet coagulation time [118], while cheese formed using milk with BB variant has higher yield, higher protein content and better quality compared to AB variant [201]. 

Such different effects have been found to be related to casein micelle size, where the AA variant is associated with large micelle size [196,199], and degrees of κ-CN glycosylation [115,202]. It was reported by Holland [203] that the higher the degree of glycosylation of κ-CN, the more stable the casein micelle structure, and the A variant is less glycosylated than variant B [204,205]. The longest curd firming time (k_20_) was found with the BE variant, while AB had better coagulation properties than AA [206]. Meanwhile, curd firmness (a_30_) of milk with the κ-CN EE variant was poorer than for AA milk, but the RCT of milk with the EE variant was shorter [207]. The possible reason for the enhancement effect of AB variant on milk rennet coagulation could be better fat entrapment [208] and water retention during cheese manufacture [209].

The effects of genetic variants of the main milk proteins on acid coagulation properties on Norwegian Red cows were studied by Ketto et al. [100], κ-CN was reported to be the only protein influencing acidification, where the AA genotype was associated with higher gel firming rate (GFR) and the gel made from milk with κ-CN AA was slightly firmer than of other variants. The E variant was found in milk with low gel firming rate [174,207]. 

### 5.4. Effect of β-lg Genetic Variant on Coagulation Properties 

The A and C variants of β-lg are associated with poor rennet coagulation properties [84], or may even be linked to non-coagulation [84,191], while the B variant is favourable for rennet coagulation [115,153]. The preference of the B variant may be linked to the cross-links and aggregates formed with whey proteins and proteolysis products produced by rennet, or larger casein micelle size [192]. In other studies, the heterozygotic genotype AA has been found to be associated with better coagulation properties than AB, and they both are more favourable for rennet coagulation than the BB variant [206]. Jensen et al. [115] reported that the AB variant of β-lg was found in both good and poorly coagulating milk in Holstein-Friesian and Jerseys cows, while, in Norwegian Red cows, AB variant was found to be associated with shorter k_20_ and higher a_30_ values than BB and AA variants [100,210]. Oloffs et al. [165] reported that variant BC was unfavourable for both RCT and a_30,_ but no relationship has been found between β-lg genotypes and RCT in Swedish Red breeds [191]. 

### 5.5. Effect of Composite Genotypes on Coagulation Properties 

The composite genotype of β-κ-CN is found to have a stronger relationship with rennet coagulation properties than single protein genotypes [6,101,105]. The most favourable milk for rennet coagulation is found to contain A_1_B-AB, A_2_B-BB and A_2_B-AB in Italian Holstein cows [105]. Heck et al. [101] reported that better cheese-making properties were associated with haplotype A_2_B of β-κ-CN in Dutch Holstein-Friesians. Meanwhile, the composite A_2_A_2_-AA, leading to low κ-CN content [10], and composites A_2_A_2_-AA, A_1_A_2_-BE and A_1_A_2_-AE, were found to be associated with poor coagulation or non-coagulating properties [10,83,103,105,199,211]. 

Milk with the composite genotypes BC-A_2_A_2_-BB and BB-A_1_A_2_-AA of α_S1_-β-κ-CN have better rennet coagulation properties than BB-A_2_A_2_-BB, BB-A_1_A_2_-BE and BB-A_2_A_2_-AA [100,105,175], and the predominant composite genotype BB-A_2_A_2_-AA is mainly found in poorly coagulating milk and non-coagulating milk [84]. This may be linked to casein micelle size [100]. However, milk with variant BB-A_2_A_2_-AA has the best acid coagulation properties among all the composite genotypes [100]. 

## 6. Heat Stability

Heat treatment is one of the most common methods employed to sterilize milk, prolong shelf-life and allow milk to be transported more easily [212]. However, some side effects can occur during heat treatment, i.e., gelling or coagulation during processing, or thickening during storage, and thus, the exploration of heat stability of milk is important in the food industry [212].

**Figure 1:**
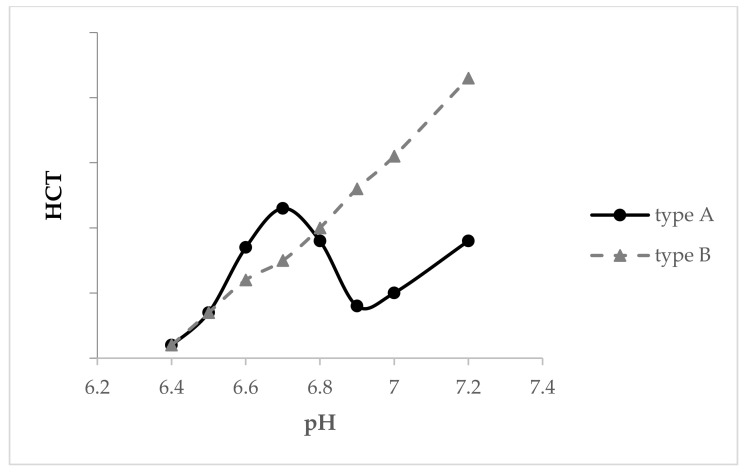
Typical type A and Type B HCT-pH profiles of milk [adapted from ref. [212].

Heat stability testing can be carried out by the observation of milk gelation or coagulation during heating at 140 °C using an oil bath, and the heat coagulation time (HCT) is related to many parameters, among which pH is the most significant [212]. The HCT-pH profiles include two regions: pH below 6.8 is the first region, while above 6.9 is the second region [213]. In general, the milk HCT-pH profile has two types, which are shown in Figure 1; type A milk has a peak at pH 6.7 and a minimum at pH 6.9, after which the curve goes up again [212], as protein charge increases and the ionic calcium activity decreases [213]; while type B milk is less stable than type A milk at pH 6.7 but more stable at pH 6.9, and its stability increases as a function of pH [212]. 

However, type A milk can be converted to type B by decreasing temperature, i.e., heating at 120 °C; adding κ-CN or some additives, i.e., oxidizing agents, removal of whey protein, or reduction in soluble salts [212].

The concentration of β-lg and κ-CN influence the HCT-pH profile significantly [214], and β-lg is the most important protein for developing Type A milk HCT-pH profile (Figure 1) [212], while Type A milk could be converted to Type B (see Figure 1) by increasing κ-CN content, as this enhances overall milk heat stability [215]. Type B curves are found to be associated with κ-CN B variant, as well as the composite genotype AB-BB of κ-CN-β-lg [216]. 

Heat-stable milk is found to be associated with the B allele of κ-CN at milk’s natural pH [217], and milk with variant BB is reported to be the most heat-stable at pH > 6.7 [216]. Milk containing the AB variant of κ-CN has longer HCT_max_ compared to AA variant, and the composite BB-AB genotype of κ-CN-β-lg, is associated with more heat-stable milk compared to AA-AA, at the pH of HCT_max_ [216]. Milk containing β-lg variant B has shorter HCT_max_, but longer HCT_min_, compared to the A variant, as the A variant has greater negative charges [23]. However, this effect is only found when the variant of κ-CN is AA, and no obvious effect of β-lg genotypes are noted with κ-CN AB and BB variants [216]. 

In the study of Keppler et al. [218], milk heat stability was determined by the unfolding temperature of the heat liable methyl group and the aromatic group regions, and maximum visible unfolding temperature. In comparison to B and C variants of β-lg, the structure of variant A changes at lower temperature, and variant C is the most stable [218]. The significant stability associated with β-lg C is suggested to be due to a stabilizing salt bridge His_59_ [129]. Heat stability of milk with different β-lg variants is associated with self-association properties, which are in the order C >> B > A [219,220,221]. When the environment becomes more acidic, β-lg A forms dimers initially and then forms octamers, while the B and C variants only form dimers due to their higher stability constants [28,222,223]. However, Hill et al. [220] and Manderson et al. [224] reported that the B variant of β-lg is less stable than the A variant. 

In other studies, no effects of β-lg or κ-CN genotypes on milk heat stability were found [225,226,227].

In addition, differences in heat stability have been found between breeds, where preheated concentrated milk from Jerseys cows is more heat-stable than that from Friesian cows [217]. 

## 7. Emulsifying and Foaming

Some functional properties of protein are based on physicochemical interactions of different components in food systems, and those related to interfacial reactions have been commonly studied [228], such as emulsifying and foaming properties [229]. 

Emulsions are defined as complex colloidal systems at a molecular level, containing two immiscible phases, such as oil and water, one of which is dispersed in the other [229]. To form an emulsion, external energy is essential for the creation of new interfacial areas, and a surfactant is needed to decrease the surface tension [230]. Differing from emulsions, which have a structure-forming unit to create structure with other food ingredients, foams are much less stable and more difficult to keep in any defined status [230]. As a result, foaming is typically the final processing step of food manufacturing [230].

### 7.1. Effects of Protein Genetic Variants on Emulsifying Properties

β-CN is a flexible and amphiphilic molecule, with a hydrophilic N-terminal, and many hydrophobic residues [231], which makes it an ideal emulsifier. It can absorb and stabilize on a newly formed oil/water interface rapidly [232], and the phosphoseryl residues clustered in its N-terminal are beneficial for emulsion formation and stability [233]. 

The most common variants, A_1_, A_2_, and B, of β-CN show different emulsifying abilities [152]. The differences are associated with pH [234], where pI of β-CN variants was in the order B (4.98) > A_1_ (4.90) > A_2_ (4.76) [152]. Thus, for illustration, when pH is at 6.7, the A_2_ variant is more soluble than A_1_ and B, and ultimately reaches the oil droplet surface more rapidly [152]. Although variant A_2_ is more efficient in emulsion formation, its emulsions are less stable than that those formed with the A_1_ and B variants, and emulsions formed by the B variant are the most stable among the three variants [152]. The maximum surface load is associated with emulsion stability; B, as the most stable variant and has a greater surface load compared to A_1_ and A_2_, while the least stable variant, A_2_, has the lowest maximum surface load [152]. The primary structures of β-CN A_1_, A_2_ and B variants are different, where the presence of an additional proline in A_2_, which increases the content of polyproline-II helix, may influence the emulsifying properties [136,152]. The net charge differences among A_1_, A_2_ and B variants, where B has one or two more positive charges than A_1_ or A_2_, respectively, leads to structural differences as well, where those extra charged residues of B could bind with other functional groups to stabilize its structure [152]. In addition, the A_1_ and B variants have more ordered structure in the absorbed state than A_2_, which also contributes to differences in their emulsifying ability [152].

### 7.2. Effects of Protein Genetic Variants on Foaming Properties

With its good interfacial behaviour, β-CN has a major influence on foaming properties of milk, and its foamability is determined by the absorption rate of protein at liquid-gas interface [235]. The foaming properties are reported to vary between genotypes, but findings are controversial. 

Ipsen and Otte [236] found that the β-CN A_2_A_2_ variant was associated with poorer foaming capacity compared to A_1_A_1_, which was due to a more extensive spread of β-CN A_1_ at the interface, which facilitated the more rapid formation of a coherent interfacial layer. In contrast, Nguyen et al. [90] reported that milk with β-CN A_2_A_2_ variant had better foaming properties than A_1_A_1_ milk. The opposite results may be caused by different foaming methods, where Ipsen and Otte [236] used 1% protein solutions with an Ultra-Turrax homogenizer, and Nguyen et al. [98] injected air bubbles into reconstituted milk samples with β-CN variants A_1_A_1_ or A_2_A_2_. 

In addition, Ipsen and Otte [236] reported that foam created by β-lg is the most stable, whereas that by α-lac had low volume and is unstable. In comparison to β-lg A variant, the B variant forms a strong interfacial layer more rapidly, and thus is associated with better foaming properties [236].

## 8. Conclusions

Studies on the frequency of casein and whey protein genetic variants, and the differences in protein structure between variants have been discussed in detail, as well as the effects of variants on milk production and composition. The contribution of milk composition, casein micelle size and genetic variants, the correlation between casein micelle size and variants on milk coagulation have also been reviewed. 

The effects of milk protein genetic variants on milk physio-chemical properties and several functionalities, including rennet coagulation and acid coagulation properties, heat stability, creaming properties, foaming properties, and possible effects on proteolysis, remain active topics of research, particularly in terms of guidance for milk selection for specific applications. Milk yield, fat and protein yield have been found to be significantly affected by β-CN genotype, while protein content (in percentage) and casein content are affected by α_S1_-CN and κ-CN genotypes. Milk coagulation properties are influenced by genotypes of α_S1_-CN, β-CN, κ-CN, β-lg and their composites, while the effects of genetic variants on heat stability have been found to be associated with κ-CN and β-lg only. 

Limited studies and research have focused on the association between α_S2_-CN genotype and milk physio-chemical and functional properties; thus, these have not been discussed in detail in this review. Studies on the effects of protein genetic variants on heat coagulation are not as extensive as those on rennet and acid coagulation properties, as are on emulsifying properties.

Cheese-making might be the most popular application in relation to milk coagulation properties, while processing at high temperatures would benefit by selection of milk with high heat resistance. However, the effects of genetic variants on milk foaming properties, increasingly of interest by users such as coffee shops, remain to be confirmed. 

It should also be noted that, rather than focusing broadly on the processibility or functional properties, milk can be selected for specific applications. For instance, milk with β-CN A_2_ variant is undesirable in cheese-making, but the weak gel it forms is more digestible and is better for making yogurt, which can be an advantage for particular markets. These findings can inform the direction for further study in relevant research areas.

## Figures and Tables

**Table 1:** Techniques for genetic profiling of milk proteins.

Method	Description	Example
Electrophoresis	Distinguishing variants from protein level depending on their electrophoretic mobility [6].	Isolation of α_S1_-CN variants [20,24,25], β-lg variants [26,27,28].
IEF (isoelectric focusing)	Determining variants by their pI [6]. Cheap and efficient [6]. Analyzing cow breeds and population from phenotypic level [6]. Recommended method to investigate biodiversity [6]	Separating β-lg H variant from B [29,30].
DNA sequencing	Analyzing proteins from genome level by scanning chromosome regions coding for individual proteins [6]. Easy sampling, e.g., milk somatic cells [31], or samples from males and nonlactating females can all be used [6]. Contributing to the identification of mutation on protein sequencing, and isolating synonymous proteins, as well as determining genetic markers [6].	PCR-RFLP [32] and direct sequencing for κ-CN [33], allele-specific-PCR [34] and PCR-single-strand conformation polymorphism for β-CN [35].
HPLC chromatography with mass spectrometry	Identify and quantify genetic variants [36,37,38].	Identification of β-CN F and G alleles [39,40].
Microarray technology	Identifying genetic variants by DNA [31], hybridization and hybridization plus enzymatic processing [41,42,43]. Analyzing single nucleotide polymorphisms (SNP) of milk proteins to confirm their genotypes, optimal probe is needed for each SNP [31].	Distinguishing κ-CN variants [31].

**Table 2:** Establishment of main protein genotypes in bovine milk.

Protein	Genotype	Methodology	Date
β-lg	Variant A, variant B	Electrophoresis	1958 [26], 1959 [27], 1961 [28]
	Variant C	Electrophoresis	1962 [44]
	Variant D	-	1966 [45]
	Variant E, variant F, variant G	Electrophoresis	1957 [11], 1963 [53] 1970 [49], 1973 [51], 1976 [52], 1981 [50]
	Variant H	IEF-IPG	1988 [29,30]
	Variant W	chromatofocusing	1990 [54]
	Variant I, variant J	Ion-exchange chromatography	1996 [55]
α-lac	Variant A, variant B	Electrophoresis	1963 [58,59]
	Variant C	Electrophoresis	1981 [60]
α_S1_-CN	Variant A, variant B, variant C	Electrophoresis	1962 [24,25]
	Variant D	Electrophoresis	1965 [20]
	Vaiant E	Electrophoresis	1963 [53], 1971 [64], 1976 [52]
	Variant F	pI	1993 [65]
	Variant G	Endonucleases	1992–1994 [66,67,68]
	Variant H	pI	1999 [69]
	Variant I	IEF, PCR	2009 [14]
α_S2_-CN	Variant A, variant B, variant C, variant D	Electrophoresis	1984 [23]
β-CN	Variant A, variant B, variant C	Electrophoresis	1961 [70], 1963 [71], 1964 [72]
	Variant A_1_, variant A_2_, variant A_3_	Electrophoresis	1966 [73,74]
	Variant D	Amino acid composition	1969 [75]
	Variant E	-	1972 [76], 1974 [77]
	Variant A_4_	Electrophoresis	1981 [60], 1995 [82]
	Variant B_Z_ (special case)	Peptide profiling	1970 [21]
	Variant F, variant G	RP-HPLC	1995 [39], 1998 [40]
	Variant H_1_	Electrophoresis, PCR	2000 [80]
	Variant H_2_	LC-MS	2002 [81]
	Variant I	PCR	2002 [83]
κ-CN	Variant A, variant B	Electrophoresis	1966 [86], 1975 [87]
	Variant J	RP-HPLC	1999 [69]
	Variant B_2_	Nucleotide sequencing	1987 [90]
	Variant C, variant E	RP-HPLC	1993 [91]
	Variant F_1_	PCR	1992 [15]
	Variant F_2_	PCR	1996 [16]
	Variant G_1_	IEF	1996 [17]
	Variant G_2_	PCR	1996 [92]
	Variant H, Variant I	DNA sequencing	1999 [93]

## Data Availability

Not applicable.
